# Optimization of Cholesterol-Loaded Cyclodextrin Combined with Soybean Lecithin as a Cryoprotectant for Rooster Sperm

**DOI:** 10.3390/vetsci11120647

**Published:** 2024-12-13

**Authors:** Mengqian He, Jiehuan Xu, Lingwei Sun, Caifeng Wu, Shushan Zhang, Jun Gao, Defu Zhang, Jianjun Dai

**Affiliations:** 1 Shanghai Municipal Key Laboratory of Agri-Genetics and Breeding, Institute of Animal Husbandry and Veterinary Science, Shanghai Academy of Agricultural Sciences, Shanghai 201106, China; hemengqian@saas.sh.cn (M.H.); jiehuanxu@saas.sh.cn (J.X.); sunlingwei@saas.sh.cn (L.S.); 20150646@saas.sh.cn (C.W.); zhangshushan@saas.sh.cn (S.Z.); gjsaas@gmail.com (J.G.); zhangdefu@saas.sh.cn (D.Z.); 2 Key Laboratory of Livestock and Poultry Resources (Pig) Evaluation and Utilization, Ministry of Agriculture and Rural Affairs, Shanghai 201106, China

**Keywords:** rooster, semen cryopreservation, CLC, SL, artificial insemination

## Abstract

There are many applications of soybean lecithin (SL) and cholesterol-loaded cyclodextrin (CLC) in sperm freezing processes. To the best of our knowledge, there have been few cases of the combined use of SL and CLC in freezing rooster semen. We investigated the effects of CLC, SL, and their combination on rooster sperm cryodamage. Three experiments were conducted: experiment 1, SL (0.5%, 1%, 1.5%, 2.0%); experiment 2, CLC (1.25 mg, 2.5 mg, 5 mg, 10 mg, 15 mg); and experiment 3, CLC + SL (2.5 mg + 0.25%, 2.5 mg + 0.5%, 2.5 mg + 1%, 2.5 mg + 1.5%). Semen samples were cryopreserved using a programmed cryostat, followed by the determination of post-thaw sperm quality, antioxidant indices, and hatching. The results showed that the combination of 2.5 mg CLC + 0.5% SL had the most significant synergistic effect on cryodamage, and the viability (56.69%), motility (54.35%), mitochondrial activity (54.23%), plasma membrane integrity (53.52%), acrosome integrity (54.71%), and antioxidant activity (MDA concentration: 5.65 nmol/mL; SOD activity: 152.73 U/mL) were significantly greater than those of the other combinations (*p* < 0.05). Nevertheless, the combined CLC and SL addition group did not substantially increase the fertilization and hatching rates of frozen semen compared with the addition of 2.5 mg CLC. In conclusion, the addition of 2.5 mg CLC and 2.5 mg CLC + 0.5% SL enhanced the quality and fertility of frozen rooster sperm.

## Simple Summary

Rooster sperm cryopreservation is essential for the preservation of biological germplasm resources and is one of the primary methods for the collection and conservation of poultry genetic resources. However, the problems of low post-freezing viability, short survival time, and low fertilization rate after artificial insemination of rooster sperm have been restricting its application in reproductive production, mainly due to cold shock and oxidative stress damage during freezing, which results in sperm plasma membrane as well as sperm damage. In this experiment, the antioxidant effect of soy lecithin and the lipid compensation effect of cholesterol on the cytoplasmic membrane were utilized to investigate the impact of adding different concentrations of soy lecithin and cholesterol and the combined effect of the two on the quality of rooster spermatozoa after freezing as well as on the hatching rate.

## 1. Introduction

The Xiayan chicken, named after its origin in rural Xiayan, Guangxi Zhuang Autonomous Region, is a specialty of Guangxi and a national geographical indication product of China. Xiayan chickens, which are known for their appearance, tender meat, and tolerance to roughage, are characterized by yellow feet, beaks, and feathers. Cryopreservation and artificial insemination of rooster semen have broad application prospects and research value in preserving and promoting this local poultry germplasm resource. Semen freezing is a cryopreservation technology that protects the genetic resources of both poultry and livestock. This method involves the use of dry ice, liquid nitrogen, or other refrigeration equipment as cold sources to store semen at low temperatures after pretreatment. This method suspends sperm metabolism, keeps the sperm dormant, and maintains its ability to fertilize after warming to an appropriate temperature. Cryopreservation is widely used in the conservation and commercialization of germplasm resources of many species. Sperm freezing technology uses males with good heredity, which is a critical process for conserving poultry genetic resources. Compared with mammals, the tails of poultry sperm are slender [1]. In addition, the composition of the plasma membrane of avian spermatozoa differs from that of mammals in that avian spermatozoa contains more unsaturated fatty acids, it contains less protein at physiological temperatures, a lower cholesterol/phospholipid ratio, and greater overall mobility [2,3]. Freezing can cause irreversible damage to sperm cell membranes. Therefore, it is essential to optimize rooster semen cryopreservation protocols to minimize the damage to rooster sperm cells during freezing.

The sperm cell membrane is comprised of lipids, mainly cholesterol (CHOL), phospholipids, and proteins. CHOL is a crucial component of the cell membrane and accounts for more than 20% of plasma membrane lipids. Moreover, CHOL plays multiple roles as a regulator of membrane function [4] and has a particular effect on cell membrane fluidity and osmotic pressure. Studies have shown that CHOL maintains the order of cells at low temperatures, prevents the formation of liquid crystals, reduces the temperature converted by the membrane, and maintains membrane flow, thus reducing cryodamage from exposure to low temperatures [5]. The leakage of phospholipids and CHOL from the semen membrane during freezing is one of the main reasons for low viability and short survival time of sperm after freezing [6]. During cryopreservation, 50% of porcine semen loses approximately 28% of its total CHOL, which causes a decrease in sperm viability, resulting in sperm inactivation. The level of CHOL is strongly associated with the maintenance of cell integrity and sperm capacitation [7]. The addition of cholesterol to bull semen diluents has been shown to effectively improve survival after freezing [8]. Napapach et al. [9] showed that adding 1 mg/mL of cholesterol-loaded cyclodextrin (CLC) to a freezing diluent improved the quality of frozen rooster semen. Additionally, Ushiyama et al. showed that loading sterols into rooster semen before cryopreservation enhanced its quality by inhibiting early apoptosis and spontaneous acrosome reactions [10]. Adding cholesterol to basic semen diluents improves the plasma membrane density and viability of cryopreserved sperm [11].

Egg yolk has been used for sperm cryopreservation in many animals [12]; however, egg yolk reduces the fertilizing ability of avian sperm, and a large body of research suggests that egg yolk reduces the respiration rate and fertilizing ability of rooster sperm [13,14,15]. Soybean lecithin (SL) is safer than egg yolk in terms of biosafety and has been reported to have no cytotoxic effects [16] or negative impact on sperm viability [17]. In roosters, 1% SL can be used instead of egg yolk to increase the freezing efficiency of frozen semen [18]. The addition of SL to different animals produces different results. In bovine semen freezing, the addition of 1.5% soy lecithin significantly improved the results of bull semen freezing. In a study related to goat semen freezing, the addition of 2% soy lecithin to Tris was shown to significantly improve the quality of goat semen after freezing [19]. In the results of a recent study [20], it was shown that the addition of 1% SL + 4.0 g/L CLC to goat semen freezing improved the efficiency of goat semen freezing, increasing the conception rate after artificial insemination. SL and CLC are considered non-animal-derived additives in semen diluents, their effects and combined application in rooster semen cryopreservation remains to be explored.

This study aimed to assess the effects of SL and CLC on motility, viability, mitochondrial activity, membrane integrity, and lipid peroxidation in Xiayan frozen–thawed rooster spermatozoa. Moreover, the combined effects of SL and CLC on frozen semen quality and fertility potential have not been well assessed in poultry. This study provides a scientific basis for the genetic breeding of chickens.

## 2. Materials and Methods

### 2.1. Chemicals

Unless otherwise stated, all chemicals used in this study were purchased from Sigma-Aldrich (St. Louis, MO, USA).

### 2.2. Animal Management and Semen Collection

The ARRIVE guidelines were used in this study. Eighteen adult Xiayan roosters were housed for 22 weeks in individual cages at 20 °C with a 14 L:10 D photoperiod. The roosters were fed a commercial feed comprised of crude protein (10%), calcium (0.9%), 3170 kcal ME/kg, and available phosphate (0.45%). The roosters had ad libitum access to water. Each rooster was housed individually in a cage (60 cm × 60 cm × 75 cm). The roosters were trained to semen collection for 3 weeks, and then semen was collected.

Semen without fecal contamination were collected in 2 mL centrifuge tubes using the dorso-abdominal massage method [21] and brought back to the laboratory at 37 °C within 30 min. Sperm concentrations were calculated using computer-assisted sperm assessment (CASA) software (CASA, IVOS Sperm Analyzer, Hamilton Thorn, Beverly, MA, USA). Samples of semen that met the criteria of the standard (>0.2 mL volume, ≥4 × 10^9^ semen/mL concentration, ≥80% sperm motility, and ≤10% abnormal morphology) were accepted for further processing [18].

### 2.3. Semen Processing

Lake’s diluent (Control): A total of 0.08 g of magnesium acetate tetrahydrate (3.73 mM), 1.92 g of sodium glutamate monohydrate (100.47 mM), 0.80 g of glucose (23.37 mM), 0.50 g of potassium acetate anhydrous (50.94 mM), and 0.30 g of polyvinylpyrrolidone (187.03 mM) were used for the control group, which was combined with 100 mL of double-distilled water (with 411 mOsm/L osmolality, pH = 7.0).

Different concentrations of CLC and SL, or their combinations, were added to the Lake’s diluents as experimental groups.

The freezing diluents: 6% dimethylacetamide (DMA) to Lake’s diluent.

Commercial SL (Type II-S, 14–23% pure; Beijing Solarbio Science &Technology Co., Ltd., Beijing, China, CAS:L8050) was added to Lake’s extenders and dissolution was accelerated with a sonicator. After stirring with a blender for 10 min and centrifuging at 5000 r/min for 15 min, the supernatant solution was passed sequentially through 1.0 μm and 0.45 μm filters [21]. The concentrations used were 0.25%, 0.5%, 1%, 1.5%, and 2%.

CLC was prepared according to previously described protocols [22,23]. CLC was prepared by adding 47.0 mg of cholesterol to 1.0 g of methyl b-cyclodextrin. Briefly, β-cyclodextrin was completely and fully dissolved in a methanol solution (1:2 (g/mL)) and cholesterol was completely dissolved in chloroform (1:5 (g/mL)). The cholesterol solution was aspirated to the β-cyclodextrin solution and mixed well (0.225:1 (*v*/*v*)). After being fully incorporated, it was poured into clean and sterile glass Petri dishes and placed in an oven at 37 °C after drying. The dried powder was β-cyclodextrin cholesterol inclusion (CLC). A working solution with CLC was prepared by adding 0, 1.25 mg, 2.5 mg, 5 mg, 10 mg, or 15 mg of CLC to 10 mL Lake’s diluent.

The effect of SL and CLC inclusion into the freezing diluent on the quality of cryopreserved semen was tested. In experiment 1, SL inclusion into Lake’s diluent was tested at the following rate: 0.25%, 0.5%, 1%, 1.5%, 2%. In experiment 2, CLC inclusion was tested at the following rate: 1.25 mg, 2.5 mg, 5 mg, 10 mg, 15 mg. In experiment 3, the inclusion of 2.5 mg CLC combined with different inclusion rate of SL (0.25%, 0.5%, 1%, 1.5%) was tested. The treatments used in the different experiments are reported in Table 1. Moreover, in experiment 4, AI was used to assess in vivo fertilizing ability and hatchability of semen cryopreserved with 1% SL, 2.5 mg CLC, and 0.5% SL combined with 2.5 mg CLC.

Pooled semen was divided into several aliquots and diluted using a two-step process. A preheated extender was added to the semen at a ratio of 1:2, and the semen was then wrapped in 10 layers of gauze and equilibrated at 4 °C for 1 h [6]. Freezing diluent precooled to 4 °C was added at a ratio of 1:1, and the mixture was further equilibrated at 4 °C for 10 min. The equilibrated semen was aspirated into 0.5 mL plastic straws.

### 2.4. Semen Freezing and Thawing

The 0.5 mL plastic straws were placed into a programmed cryometer (Ice Cube 14 M, Sylab) to cool the temperature at a rate of 7 °C/min from 4 °C to −35 °C and 60 °C/min from −35 °C to −140 °C. The straws were rapidly immersed in liquid nitrogen and stored until further use.

During sperm thawing, a 0.5 mL plastic straw containing semen was removed from the liquid nitrogen and immediately placed in a water bath at 37 °C for 30 s [24]. When the sperm had completely dissolved in the plastic straw, the circumference of the tube was dried with a paper towel, the sides of the tube were then cut with scissors and the sperm was transferred to a 2.0 mL centrifuge tube. Various measurements were performed after thawing.

### 2.5. Computer-Assisted Sperm Analysis

A 10 µL sample of semen was placed on a warm (37 °C) glass slide for examination and the motility characteristics of the semen were assessed both before and after freezing using CASA. Ten randomly selected fields containing at least 200 spermatozoa were examined at 400× magnification using a phase-contrast microscope.

### 2.6. Semen Membrane Integrity

Sperm membrane integrity was tested using SYBR-14 and propidium iodide kits (L7011; Invitrogen, Thermo Fisher Scientific, Waltham, MA, USA) [22], which use a fluorescent staining approach to distinguish between live and dead spermatozoa. To stain the sperm suspension, 300 μL was combined with 5 μL of SYBR-14 solution and incubated at 24–27 °C for 10 min. Then, 5 μL of propidium iodide was added and stained for 5 min. Spermatozoa were collected. At least 200 spermatozoa were observed by fluorescence microscopy. Membrane integrity was calculated as the percentage of undamaged plasma membranes.

### 2.7. Sperm Acrosomal Integrity

Giemsa staining was used to determine sperm acrosomal integrity [25]. After thawing, sperm smears were prepared and air-dried. Sperm were fixed with formaldehyde for 15 min and rinsed. Fixed sperm were stained with Giemsa solution for 12 h, rinsed, air-dried, and examined under a microscope. Three microscopy fields with at least 200 sperm in each sample were observed, and the acrosomal integrity rate was calculated. Those with normal appearance or slight acrosomal expansion were considered intact acrosomal sperm, whereas those with moderate or severe acrosomal expansion or deficiency were considered sperm with malformed acrosomes [26].

### 2.8. Determination of Antioxidant Indices

Malondialdehyde (MDA) levels were measured using the thiobarbituric acid reactive substance (TBARS) test. After thawing, the sperm concentration was adjusted to a final concentration of 5 × 10^6^ mL^−1^. Briefly, 1 mL of 15% (*w*/*v*) trichloroacetic acid was added to each tube, followed by 1 mL of 0.375% (*w*/*v*) thiobarbituric acid. The tubes were vortexed and boiled in a water bath for ten minutes. The samples were then removed from the boiling water and placed in an ice box to stop the process. Finally, the supernatant was collected and the absorbance at 532 nm was determined using a Shimadzu UV 2100 spectrophotometer (Shimadzu, Kyoto, Japan). The concentration of MDA was measured using a standard curve with a known value (0.5–32 µM) [27].

A SOD assay kit (Nasdox™-Superoxide Dismutase Assay Kit, Navand Salamat Company, Urmia, Iran) and the nitro blue tetrazolium reduction method were used for the evaluation of SOD activity, which was expressed as U/mL. The samples’ concentrations (U/mg) were obtained by measuring the mean absorbance values using a spectrophotometer at 630 nm, and the SOD activity was calculated using a standard calibration curve.

### 2.9. Sperm Mitochondrial Activity

Rhodamine (Rh123) staining (100 nmol/L) [28] was used to detect mitochondrial activity. The semen samples were kept at 37 °C for 30 min in the dark. Green fluorescence in the sperm tail is considered indicative of high mitochondrial activity. At least 200 sperm were observed in three different microscopic fields, and the number of sperm with high mitochondrial activity was calculated.

### 2.10. Artificial Insemination

To evaluate reproductive performance, 40 Xiayan hens (28 weeks old) were randomly divided into four groups (each hen housed in individual battery cages measuring 70 cm × 70 cm × 85 cm^3^) (n = 10 in each group) and artificially inseminated with cryo-thawed sperm containing Lake’s diluent, 0.25% CLC, 1% SL, and 0.25% CLC + 0.5% SL. All hens underwent intravaginal insemination according to the following protocol: sperm transfusion was completed within half an hour of thawing, 0.20 mL (100 × 10^6^) spermatozoa were used for insemination. AI was performed twice a week for 1 month. All insemination processes were performed between 15:00 and 16:00. Egg collection began 2 days after artificial insemination, and eggs were collected for 6 consecutive days. The eggs were then numbered by hen number and stored for incubation at 37 °C and 75% humidity to assess fertility and hatchability. In each group, 50 eggs were placed on a rotating tray in a common incubator for 21 d. 

On day 9 of incubation, fertilization rate (%) = n. embryonic eggs/total eggs set*100, and, on the 21st day, hatchability (%) = n. viable chicks/total egg set*100 and hatchability on fertile eggs (%) = n. viable chicks/fertilized eggs+100 were evaluated.

### 2.11. Statistical Analysis

The freezing experiments were replicated five times, and the fertilization experiment was replicated three times. Each replicate of each experiment used a new pool. Data were analyzed using IBM SPSS Statistics (version 20.0; SPSS Inc., Chicago, IL, USA). All data were examined using the Shapiro–Wilk test and found to have a normal distribution. The groups were compared using one-way ANOVA, post hoc analysis, and the least significant difference test. The differences between the two groups were evaluated using the Student’s *t*-test. The significance threshold was set at *p* < 0.05. Data is reported as mean ± SEM.

## 3. Results

Fresh sperm from the ejaculate had an average volume of 1.20 ± 0.26 mL, a concentration of (742.68 ± 32.12) × 10^6^ semen/mL, and a pH of 6.19 ± 0.18. Total sperm motility exceeded 80%, viability exceeded 90%, and sperm deformities occurred at a rate of 7%.

### 3.1. Experiment 1: Effect of Cholesterol on Cryopreserved Xiayan Rooster Sperm

**Figure 1:**
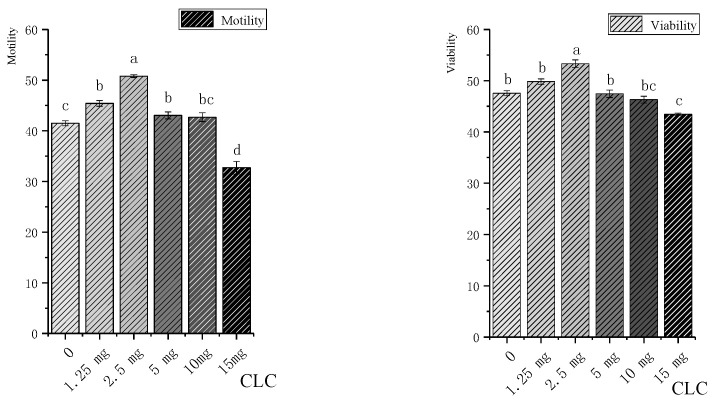
Effects of different CLC concentrations on the motility and viability of cryopreserved Xiayan rooster sperm. Note: lowercase letters indicate significant differences between treatments (*p* < 0.05). “a, b, c, bc”—Values with different letters in the figure indicate significant differences (*p* < 0.05).

The motility and viability of rooster sperm treated with different concentrations of CLC cryoprotectant are shown in Figure 1. After thawing, the motility (50.78%) and viability (54.53%) of the 2.5 mg CLC-supplemented group were significantly higher than those of the other groups (*p* < 0.05). With a further increase in CLC concentration, the sperm motion parameters gradually decreased and reached a minimum (*p* < 0.05) at a CLC concentration of 1.5%. The addition of 2.5 mg of CLC increased the sperm’s post-freezing viability by 10% compared to the control group.

The mitochondrial activity, plasma membrane integrity, acrosome integrity, and antioxidant ability of frozen rooster semen treated with different CLC concentrations are shown in Table 2. The mitochondrial activity of 2.5 mg CLC and 5 mg CLC was higher than the other groups (2.5 mg vs. 5 mg, 49.29% vs. 47.11). The plasma membrane integrity (2.5 mg, 48.23%), and acrosome integrity (2.5 mg, 49.51%) of the 2.5 mg CLC group were the highest after thawing and were much more significant than those of the control group (42.14%, 37.57%, and 39.18%, *p* < 0.05). With increasing CLC concentration, gradual decreases were observed in the mitochondrial activity, plasma membrane integrity, and acrosome integrity of frozen sperm. When the CLC concentration was 2.5 mg and 5 mg, the MDA content was the lowest (2.5 mg vs. 5 mg, 6.47 nmol/mL vs. 6.73 nmol/mL) and the SOD activity was the highest (2.5 mg vs. 5 mg, 124.41 U/mL vs. 122.88 U/mL), which was significantly different from that of the control group (7.10 nmol/mL, 115.57 U/mL) (*p* < 0.05). A further increase in CLC concentration did not improve the antioxidant capacity of frozen rooster sperm.

### 3.2. Experiment 2: Effect of Soybean Lecithin on Cryopreserved Xiayan Rooster Sperm

**Figure 2:**
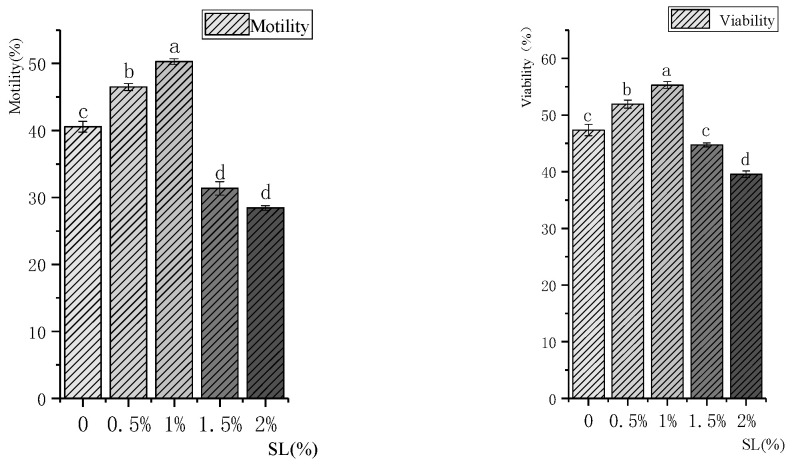
Effects of different concentrations of SL supplementation on the motility and viability of cryopreserved Xiayan rooster sperm. “a, b, c, d”—Values with different letters in the figure indicate significant differences (*p* < 0.05).

The motility and viability of rooster semen treated with different concentrations of SL after freezing and thawing are shown in Figure 2. The quality of thawed sperm in the 1% SL-supplemented group was the highest. Compared with the control group, sperm motility increased by about 10% (50.27% vs. 40.56%), and sperm viability increased by about 8% (55.30% vs. 47.36%) of the 1% SL group. The 1% SL was significantly higher than that of the other groups (*p* < 0.05). With a further increase in the SL concentration (≥1.5%), sperm motility and viability significantly decreased (*p* < 0.05).

The effects of SL addition on the mitochondrial activity, plasma membrane integrity, and acrosome integrity of frozen rooster sperm are shown in Table 3. The results demonstrate that the mitochondrial activity (51.12%), plasma membrane integrity (44.28%), and acrosome integrity (51.16%) of the 1% SL group were significantly higher than those of the other groups (*p* < 0.05). When the concentration of SL was ≥1.5%, the acrosome integrity of the sperm was significantly lower than that of the control group. When the SL concentration reached 2%, sperm mitochondrial activity and plasma membrane integrity significantly decreased (*p* < 0.05). The MDA content in the 1% SL group was 6.13 nmol/mL, which was significantly lower than that in the control group (7.04 nmol/L, *p* < 0.05). SOD activity in the 1% SL group was 141.14 U/mL, which was significantly higher than that in the control group (114.66 U/mL, *p* < 0.05). The antioxidant capacity of frozen rooster semen was not improved by further increasing the amount of SL, and the antioxidant capacity of frozen rooster semen with the addition of 1.5% soy lecithin was lower than that without the addition of SL.

### 3.3. Experiment 3: Effect of CLC and SL Combinations on Cryopreserved Xiayan Rooster Sperm

**Figure 3:**
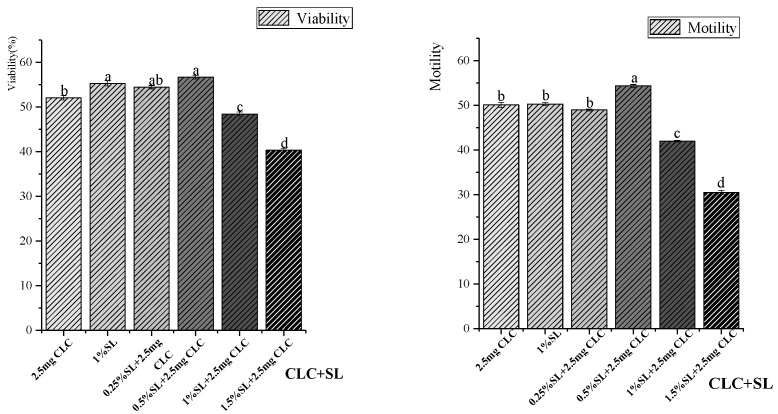
Effects of combined CLC and SL addition on the motility and viability of cryopreserved Xiayan rooster sperm. “a, ab, b, c, d”—Values with different letters in the figure indicate significant differences (*p* < 0.05).

The motility and viability of the thawed sperm are shown in Figure 3. With the addition of 2.5 mg CLC, the combined addition of 0.5% SL had a positive synergistic effect on rooster sperm cryopreservation. This resulted in the highest viability and motility (56.69% and 54.35%, respectively) in all groups, which was significantly greater than that in the 2.5 mg CLC addition group (52.07%, *p* < 0.05). However, in the presence of 2.5 mg CLC, high concentrations (1% and 1.5%) of SL significantly decreased (*p* < 0.05) sperm viability and motility.

The effects of different concentrations of CLC and SL supplementation on mitochondrial activity, plasma membrane integrity, and acrosome integrity of frozen rooster sperm are shown in Table 4. The thawed sperm in the 2.5 mg CLC + 0.5% SL supplement group had the highest plasma membrane integrity (53.52%) and acrosome integrity (54.71%), which were significantly higher than that of the other supplement groups (*p* < 0.05); mitochondrial activity in 0.5% SL + 2.5 mg CLC and 1% SL had the highest(1% SL vs. 0.5% SL + 2.5 mg CLC, 52.14% vs. 54.23%). The MDA content of this group was 5.65 nmol/mL (*p* < 0.05), and the SOD activity was 152.73 U/mg, which was significantly different from that of the other groups (*p* < 0.05). With 2.5 mg CLC addition, added SL concentration reached 1% and 1.5%; the combined SL addition had a negative effect on rooster semen freezing, which was lower than that observed in the absence of SL. Further increasing SL addition did not improve the antioxidant capacity of frozen rooster sperm. The antioxidant capacities of the 1.0% (6.85 nmol/mg vs. 117.44 U/mL, *p* < 0.05) and 1.5% (7.45 nmol/mL vs. 109.33 U/mL, *p* < 0.05) SL addition groups were lower than those of the control groups.

### 3.4. Experiment 4: Hatching Evaluation of Cryopreserved Xiayan Rooster Sperm

Table 5 shows the effects of SL, CLC, and their combination on the reproductive performance of cryopreserved Xiayan rooster sperm. The results showed that the application of 2.5 mg CLC and 1% SL as sperm cryoprotectants significantly increased egg fertilization and hatchability on egg set (*p* = 0.044 and *p* < 0.01, respectively). In the combined group, egg fertilization was increased by more than 10% relative to the control group and by 7% for the 1% SL addition; however, there was no significant difference in the egg fertilization rate in the 2.5 mg CLC and 1% SL compared to the group with 2.5 mg CLC alone.

## 4. Discussion

Semen-freezing technology is particularly vital, as it is the most crucial part of artificial insemination. The problem with rooster sperm is that the best cryoprotectant for obtaining the highest post-thaw quality is glycerol, which acts as a contraceptive when the final concentration is above 0.1% by volume [29]. However, cryoprotectants are often toxic and reduce the overall sperm quality. Freezing and thawing damages the mitochondria of spermatozoa and alters the osmolarity of the sperm environment, resulting in damage to the plasma membrane and acrosomal structures of the head of the spermatozoa. This is considered an essential factor that increases the susceptibility of poultry semen to cryoinjury during the freezing process [30].

The addition of SL and CLC significantly increased sperm survival after freezing, compared to the other dilutions. Significant fertilization rates were obtained from sperm cryopreserved in SL and CLC dilutions compared with conventional dilutions. SL and cholesterol are effective in preserving sperm function.

In the present study, we found that when adding CLC alone, rooster sperm’s post-freezing quality was significantly higher than that of the control group, and the highest sperm post-freezing survival was observed when the cholesterol concentration was increased to 0.25%. CHOL is a cell membrane component that plays a crucial role in maintaining sperm membrane integrity. Studies have shown that plasma membrane damage is one of the leading causes of cell death [31,32]. Cholesterol increases sperm plasma membrane fluidity at low temperatures. The sensitivity of semen to cold stress is also reduced [32]. The results of Chuaychu [9] showed that adding 2–3 mg/mL CLC significantly increased the viability of frozen rooster semen. This study showed that the optimal CLC concentration for rooster semen cryopreservation was 0.25%.

Because CHOL itself has specific antioxidant characteristics, it has been shown that the addition of CLC to horse semen cryopreservation is effective in increasing the antioxidant capacity of spermatozoa [33]. Cryopreservation may indirectly lead to membrane damage by enhancing LPO and lead to sub-lethal damage. In the present study, the antioxidant level of spermatozoa was significantly enhanced by adding 0.25% CLC in the freezing process. Dead spermatozoa are a source of free radicals, and the addition of CLC reduces sperm death, leading to a decrease in ROS production. When the concentration of CLC is too high, the fluidity of the sperm membrane is reduced, increasing the vulnerability of the sperm to mechanical damage [23]. When cholesterol levels are too high, the fluidity of the cell membrane is significantly reduced, and sperm are more vulnerable to mechanical damage. Barrenholtz showed that CHOL inhibits Na+/K+ ATP pumps in membranes [34], which affects sperm viability. However, when the CLC content is too high, CLC is easily oxidized and produces large amounts of hydroxysterols that are toxic to spermatozoa. In the present study, sperm viability gradually decreased after freezing at CLC concentrations greater than 1%.

SL is a plant extract that causes less pollution. In addition, soybean lecithin contains not only rich lecithin, but also a large amount of phosphatidylserine (PS). PS can promote the elimination of free radicals, improve the antioxidant capacity, and reduce the level of endotoxin in mice [35]. When appropriate amounts of phosphatidylserine are added, it can be used as an antioxidant to reduce the damage of free radicals caused by sperm cold stress during semen freezing and improve the survival rate and motility of sperm after freezing [36,37]. Hossein et al. [38] used SL as a substitute for egg yolk in an in vitro evaluation of goat semen freezing. They showed that adding SL at a concentration of 1.5% to the basal diluent significantly improved sperm viability. Singh et al. [39] added 1.5% SL to a frozen bovine semen diluent to achieve high sperm viability after freezing. Tarig et al.’s [40] results showed that adding SL at 1.5% significantly improved the indices of bovine semen after freezing. The SL content in the semen of different animals is also different, which may be related to the differences among species. This study revealed that the optimum SL concentration for cryopreservation of rooster semen was 1.0%.

In addition, it has been shown that SL can be toxic to spermatozoa if the concentration is too high. With excessive SL supplementation, viscous aggregates are formed in the suspension after thawing, which adhere to the sperm flagellum and reduce the motility of the spermatozoa. A gradual loss of motility of bull spermatozoa with increasing SL concentration was observed in a previous study [41], and it was hypothetically ascribed to the presence of debris and higher viscosity. Excessive SL also decreased mitochondrial transmembrane sites, causing changes inside and outside the mitochondrial membrane, leading to changes in ATP synthase and other enzymes associated with driving ATP synthesis. Still, mitochondrial damage also leads to oxidative stress damage in spermatozoa [42,43].

This study investigated the effect of CLC and SL alone on the cryopreservation of rooster semen. It was found that adding the two antifreeze agents alone significantly improved the efficiency of rooster semen cryopreservation. The present results show that CLC and SL play a positive cryoprotective action and improve fertility and hatchability of frozen/thawed semen compared to the control; moreover, CLC improves hatchability/fertile eggs compared to the control. The combined addition of CLC and SL also improves the in vivo efficiency of rooster semen cryopreservation, providing results very similar to the action of CLC alone. The present results suggest a different cryoprotective action of CLC and SL. More phospholipids, antioxidant components, and unsaturated fatty acids are present in SL and further studies are needed to explore the mechanism of their action with cholesterol during the sperm freezing process.

It can be seen that the quality of the sperm itself plays a crucial role in the final fertilization and hatching rates achieved. In this experiment, 0.5% SL + 2.5 mg CLC gave the best AI results. However, there was no significant difference with the 0.25 mg CLC group. The addition of 1% SL significantly improved the AI results of the sperm compared to control group, which was attributed to the protective effect of the cryoprotectant on the sperm during the freezing process. The AI results of the 1% SL group were significantly lower than those of the 2.5 mg CLC and 0.5% SL + 2.5 mg CLC groups. This may be due to the addition of too high a concentration of SL, with some of the dense polymer suspension adhering to the sperm flagellum after thawing, resulting in blocked sperm–oocyte binding.

## 5. Conclusions

Adding 0.5% SL + 2.5 mg CLC did not significantly differ from adding 1% SL or 2.5 mg CLC alone, in terms of sperm post-freezing quality. However, the antioxidant properties were significantly better than those of the groups with 1% SL or 2.5 mg CLC alone. The addition of 2.5 mg CLC alone achieved the same effect as the combined treatment in terms of AI results.

## Figures and Tables

**Table 1:** Different basic diluent formulations.

Experiment 1 (SL)					
Formula					
SL					
0.25%	0.5%	1%	1.5%		2%
0.25 g	0.5 g	1 g	1.5 g		2 g
Diluents	Lake’s (10 mL)				
PH	7.0~7.4				
Freezing diluents	6% DMA in Lake’s				
**Experiment 2 (CL)**					
Formula					
CLC					
0.125%	0.25%	0.5%	1%		1.5%
1.25 mg	2.5 mg	5 mg	10 mg		15 mg
Diluents	Lake’s (10 mL)				
PH	7.0~7.4				
Freezing diluents	6% DMA in Lake’s				
**Experiment 3 (CLC + SL)**					
Formula					
CLC + SL					
2.5 mg CLC + 0.25% SL	2.5 mg CLC + 0.5% SL	2.5 mg CLC + 1% SL		2.5 mg CLC + 1.5% SL	
2.5 mg CLC + 0.25 g SL	2.5 mg CLC + 0.5 g SL	2.5 mg CLC + 1 g SL		2.5 mg CLC + 1.5 g SL	
Diluents		Lake’s (10 mL)			
PH		7.0~7.4			
Freezing diluents		6% DMA in Lake’s			

**Table 2:** Effects of CLC addition on the mitochondrial activity, plasma membrane integrity, and acrosome integrity of cryopreserved Xiayan rooster sperm.

CLC Concentration (mg)	Mitochondrial Activity (%)	Plasma Membrane Integrity (%)	Acrosome Integrity (%)	Oxidation Index (%)	
				MDA (nmol/mL)	SOD (U/mL)
0	42.14 ± 0.19 c	37.57 ± 0.11 c	39.18 ± 0.52 c	7.10 ± 0.23 ab	115.57 ± 0.23 bc
1.25	45.71 ± 0.31 bc	43.21 ± 0.19 b	44.73 ± 0.37 b	7.31 ± 0.11 a	117.51 ± 0.76 b
2.5	49.29 ± 0.44 a	48.23 ± 0.44 a	49.51 ± 0.42 a	6.47 ± 0.53 c	124.41 ± 0.35 a
5	47.11 ± 0.64 ab	43.38 ± 0.40 b	44.63 ± 0.95 b	6.73 ± 0.33 c	122.88 ± 1.58 a
10	45.38 ± 0.52 b	42.50 ± 0.68 b	42.64 ± 1.05 bc	7.02 ± 0.30 b	119.43 ± 1.73 ab
15	40.44 ± 0.82 c	34.15 ± 0.19 d	36.33 ± 1.64 c	7.48 ± 0.10 a	110.06 ± 1.88 d
*p* value	*p* < 0.01	*p* < 0.01	*p* < 0.01	*p* = 0.03	*p* < 0.01

“a, ab, b, bc, c, d”—Values with different letters in the same column indicate significant differences (*p* < 0.05).

**Table 3:** Effects of SL addition on the mitochondrial activity, plasma membrane integrity, and acrosome integrity of cryopreserved Xiayan rooster sperm.

SL Concentration (%)	Mitochondrial Activity (%)	Plasma Membrane Integrity (%)	Acrosome Integrity (%)	Oxidation Index (%)	
				MDA (nmol/mL)	SOD (U/mL)
0	39.66 ± 0.94 c	36.46 ± 0.87 c	38.06 ± 0.27 c	7.04 ± 0.23 c	114.66 ± 1.53 c
0.5	47.09 ± 0.92 b	40.62 ± 0.79 b	45.20 ± 1.41 b	6.59 ± 0.21 d	129.44 ± 1.71 b
1	51.12 ± 0.85 a	44.28 ± 0.89 a	51.16 ± 1.06 a	6.13 ± 0.11 e	141.14 ± 1.13 a
1.5	36.83 ± 1.12 c	36.31 ± 1.15 c	35.16 ± 1.37 d	8.11 ± 0.09 b	106.80 ± 0.97 d
2	29.85 ± 1.79 d	31.13 ± 1.02 d	29.76 ± 2.20 e	9.12 ± 0.30 a	90.99 ± 2.03 e
*p* value	*p* < 0.01	*p* < 0.01	*p* < 0.01	*p* < 0.01	*p* < 0.01

“a, b, c, d, e”—Values with different letters in the same column indicate significant differences (*p* < 0.05).

**Table 4:** Effects of different concentrations of soy lecithin and cholesterol addition on the quality of cryopreserved Xiayan rooster sperm.

Groups	Mitochondrial Activity	Plasma Membrane Integrity	Acrosome Integrity	Oxidation Index (%)	
				MDA (nmol/mL)	SOD (U/mL)
2.5 mg CLC	47.73 ± 1.64 b	48.38 ± 0.40 b	48.63 ± 0.35 b	6.71 ± 0.33 b	123.97 ± 0.93 c
1% SL	52.17 ± 0.71 a	45.03 ± 0.71 c	50.31 ± 0.44 b	6.21 ± 0.14 c	141.35 ± 0.79 b
0.25% SL + 2.5 mg CLC	48.23 ± 1.21 b	49.90 ± 0.60 b	49.44 ± 0.49 b	6.02 ± 0.28 c	144.95 ± 0.84 b
0.5% SL + 2.5 mg CLC	54.23 ± 1.21 a	53.52 ± 0.42 a	54.71 ± 0.33 a	5.65 ± 0.59 d	152.73 ± 0.65 a
1% SL + 2.5 mg CLC	37.61 ± 0.34 c	39.90 ± 0.60 c	40.44 ± 0.49 c	6.85 ± 0.48 b	117.44 ± 0.72 d
1.5% SL + 2.5 mg CLC	28.51 ± 0.55 d	27.11 ± 0.33 d	29.41 ± 0.37 d	7.54 ± 0.49 a	109.33 ± 0.39 e
*p* value	*p* < 0.01	*p* < 0.01	*p* < 0.01	*p* = 0.066	*p* < 0.01

“a, b, c, d, e”—Values with different letters in the same column indicate significant differences (*p* < 0.05).

**Table 5:** Reproductive performance of cryopreserved Xiayan rooster sperm frozen with different cryoprotectants via artificial insemination.

Treatments	Fertilized (%)	Hatchability (%)	Hatchability of Fertile Egg (%)
control	33.53 ± 2.13 c	13.66 ± 0.57 c	43.46 ± 1.98 bc
2.5 mg CLC	43.71 ± 1.56 a	23.67 ± 1.52 a	52.74 ± 2.21 a
1% SL	37.96 ± 1.64 b	16.56 ± 1.15 b	47.74 ± 2.77 b
0.5% SL + 2.5 mg CLC	44.57 ± 2.43 a	25.00 ± 2.00 a	54.43 ± 1.95 a

“a, b, bc, c”—Values with different letters in the same column indicate significant differences (*p* < 0.05).

## Data Availability

Data supporting the reported results are available within the article and upon request to the corresponding author.
